# Anti-RNApol3-Associated myocarditis: an emerging disease linking autoimmunity and infection

**DOI:** 10.1186/s13613-025-01443-1

**Published:** 2025-03-24

**Authors:** Paul Quentric, Jean-Luc Charuel, Quentin Moyon, Guillaume Hékimian, Karim Dorgham, François Lifermann, Mathieu Kerneis, Alexis Mathian, Karim Aacha, Isabelle Melki, Juliette Chommeloux, Matthieu Petit, Melchior Gautier, Pierre Bay, Philippe Rouvier, Etienne Charpentier, Omaira da Mata-Jardin, Lucie Lefevre, Christophe Parizot, Ouriel Saura, David Levy, Sofia Ortuno, Matthieu Schmidt, Charles-Edouard Luyt, Guy Gorochov, Zahir Amoura, Alain Combes, Marc Pineton de Chambrun

**Affiliations:** 1 Centre d’Immunologie et des Maladies Infectieuses (CIMI-Paris), Sorbonne Université, Inserm, Paris, France; 2 Service de Médecine Interne 2, Centre de Référence National Lupus Systémique et Syndrome des Anticorps Anti-phospholipides, Sorbonne Université, Assistance Publique-Hôpitaux de Paris (AP-HP), Hôpital La Pitié–Salpêtrière, Institut E3M, Paris, France; 3 Département d’Immunologie, Sorbonne Université, AP-HP, Hôpital La Pitié–Salpêtrière, Paris, France; 4 Service de Médecine Intensive-Réanimation, Sorbonne Université, AP-HP, Hôpital La Pitié–Salpêtrière, 47–83, boulevard de l’Hôpital, Paris Cedex, 75651 France; 5 Service de Médecine Interne, Centre Hospitalier de Dax, Dax, France; 6 ACTION Study Group, Département de Cardiologie, Sorbonne Université, AP-HP, Hôpital La Pitié-Salpêtrière, Paris, France; 7 Institut de Cardiométabolisme et Nutrition (ICAN), Sorbonne Université, INSERM, UMRS_1166-ICAN, Paris, F-75013 France; 8 Service de Pédiatrie Générale Paris, Université Paris Diderot, AP-HP, Hôpital Robert-Debré, Paris, France; 9 Service de Médecine Intensive Réanimation and UPEC, Hôpitaux universitaires Henri Mondor, AP-HP, DMU Médecine, Université Paris Est), INSERM, Unit é U955, équipe 18, Créteil, 94010 France; 10 Service d’Anatomopathologie, Sorbonne Université, AP-HP, Hôpital La Pitié–Salpêtrière, Paris, France; 11 Département d’Imagerie Cardiothoracique, Sorbonne Université, AP-HP, Hôpital La Pitié–Salpêtrière, Paris, France

**Keywords:** Fulminant myocarditis, Anti-RNA-polymerase III autoantibodies, Extracorporeal membrane oxygenation, Autoimmunity, Viral infection, Systemic sclerosis

## Abstract

**Background:**

Fulminant myocarditis (FM) is a severe condition primarily triggered by viruses. Anti-RNA polymerase III autoantibodies (RNApol3) which are typically found in patients with severe systemic sclerosis, have been reported in patients with influenza-related FM. Our objective is to provide additional insight into RNApol3-associated FM.

**Methods:**

We retrospectively included all patients admitted to our institution between January 2013 and June 2023 with acute myocarditis and positive serum RNApol3. We compared their characteristics, etiologies, and outcomes with those of a cohort of RNApol3 negative acute myocarditis.

**Results:**

Twenty-nine RNApol3-positive patients, comprising 83% females with a mean age of 39 ± 12 years, were included in this study. Each patient was admitted to the intensive care unit at least once and 11 (38%) relapsed. Triggers included influenza virus in 55% and SARS-CoV-2 virus in 48% of cases. The lowest left ventricular ejection fraction was 10 [5-10] % and the highest troponin value was 82 [22–360] times the ULN. Patients required dobutamine (94%), veno-arterial extracorporeal membrane oxygenation (85%) and pericardiocentesis (38%). At the last follow-up, 76% of patients were still alive, while 7% had undergone cardiac transplantation, and 3% required a left ventricular assist device. Compared to RNApol3-negative cases, RNApol3-positive myocarditis was associated with female gender, fulminant evolution, tamponade, a higher likelihood of being caused by a proven viral infection, and a higher rate of relapse.

**Conclusion:**

RNApol3-associated myocarditis is an emerging disease linking autoimmunity and infection and a unique cause of acquired, pathogen-specific, organ-specific immunodeficiency. RNApol3 should be screened in all cases of FM, especially in young women infected by RNA viruses. The risk of FM in RNApol3-positive systemic sclerosis needs further investigation.

**Supplementary Information:**

The online version contains supplementary material available at 10.1186/s13613-025-01443-1.

## Graphical abstract

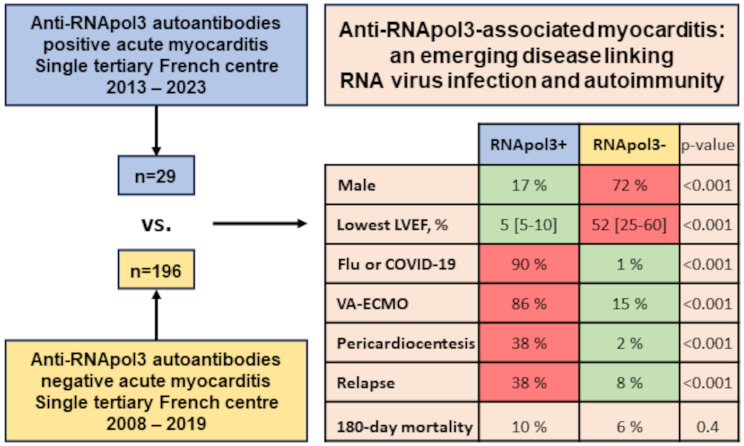

## Introduction

Fulminant myocarditis is a complex, multifaceted, and often severe disease characterized by inflammation of the myocardium, induced by viruses, bacteria, toxins, or immune-mediated conditions [1]. The etiological investigation is challenging due to the numerous diseases known to cause myocarditis [2]. Viruses are considered the leading cause, yet the pathophysiology of virus-induced myocardial lesions remains elusive. In 2020, Pineton de Chambrun and colleagues first described, in 11 patients, the unusual association of mainly influenzae virus-induced fulminant myocarditis with persistent autoantibodies targeting RNA-polymerase III (RNApol3) [3]. More recently, these rare autoantibodies have been reported in patients with severe COVID-19-related fulminant myocarditis [4]. Anti-RNApol3 autoantibodies are typically found in patients with systemic sclerosis associated with severe organ involvement, yet none of the patients in these two studies had such a condition. Systemic sclerosis is an orphan disease characterized by autoimmunity, fibrosis of the skin and internal organs and vasculopathy. It has the highest mortality among all rheumatic diseases [5]. Systemic sclerosis cardiac involvement is frequent and very heterogeneous: chronic inflammatory myocarditis, myocardial fibrosis [6, 7], congestive heat failure, arrythmia, impaired ventricular relaxation [8] and also pulmonary hypertension [9]. The laboratory diagnosis of systemic sclerosis (SSc) relies on the detection of antinuclear antibodies, primarily characterized by four mutually exclusive specificities: anti-topoisomerase I (Scl-70), anti-centromere, anti-nucleolar, or anti-RNApol3 autoantibodies. The latter are highly specific for SSc, found in approximately 5–20% of patients, and were initially proposed as markers of severe disease. RNApol3 is a protein that transcribes DNA to synthesize 5 S ribosomal RNA, tRNA, and other small RNAs. Transfer RNA is a small RNA that connects messenger RNA to the growing amino acid chain during protein synthesis. An increasing body of literature suggests that RNApol3 can transcribe viral or cellular DNA sequences into immunostimulatory RIG-I ligands. These ligands bind to RIG-I-like receptors, which are key sensors of viral infections. This interaction mediates the transcriptional induction of type I interferons and other genes that collectively orchestrate an antiviral host response [10]. The role of anti-RNApol3 autoantibodies in the pathophysiology of viral fulminant myocarditis remains to be elucidated. Given the limited data on this new disease, we aim to further describe the spectrum and outcomes of anti-RNApol3-associated myocarditis.

## Materials and methods

### Patient selection

We retrospectively included all patients admitted to our institution between January 2013 and June 2023 with myocarditis and positive serum anti-RNApol3 autoantibodies (patients were sourced from our immunology department’s database). We used the clinically suspected myocarditis classification criteria and the ESC 2015 pericarditis definition for patient inclusion [11, 12]. Myocarditis was subsequently categorized as definite, probable, and possible according to the myocarditis classification by Bonaca et al. For the diagnosis of systemic sclerosis, we applied the ACR-EULAR 2013 classification criteria [13].

### Data collection

The following information was collected on standardized forms: epidemiologic parameters; severity of the underlying condition according to the Charlson comorbidity index; medical history; myocarditis and associated infection history, manifestations and complications; day-0 Sequential Organ Failure Assessment (SOFA) score and Simplified Acute Physiology Score (SAPS) II; day-0 and in-intensive care unit (ICU) clinical and biological parameters; day-0 and in-ICU organ-failure support treatment; day-0, in-ICU and last-follow-up echocardiography parameters; in-ICU myocarditis-specific treatment; in-ICU and follow-up CT-scan and cardiac magnetic resonance imaging (CMR); systemic sclerosis-related findings; anti-RNApol3 assay result; vital status at ICU and hospital discharge, as well as at last follow-up. All outcome variables, including heart transplantation and relapse, were evaluated at the most recent follow-up for each patient.

### Anti-RNA polymerase III autoantibodies

Every patient with clinically suspected myocarditis admitted to our hospital undergoes a systematic noninvasive diagnostic workup, including laboratory analyses, imaging studies, and low-risk pathological examinations (e.g., salivary accessory gland biopsy). Screening for antinuclear antibodies and anti-RNA polymerase III autoantibodies is performed routinely on the first working day after admission as part of this workup as follows: indirect immunofluorescence assay was run on HEp-2000 cells (Immuno Concepts, Sacramento, CA, USA), when positive (≥ 1/80) and the immunofluorescence labeling pattern suggested anti-RNApol3 autoantibodies (fine-speckled nuclear-labeling pattern with small dots), a confirmatory immunodot assay (i.e. a laboratory diagnostic technique used to detect all systemic sclerosis specific auto-antibodies in the serum at the same time) all systemic sclerosis-associated autoantibodies (Euroline Systemic Sclerosis Test, Euroimmun France, Bussy Saint-Martin, France) was performed. Detection of anti-RNA polymerase III antibodies is qualitative rather than quantitative.

### Myocarditis control cohort

To compare the characteristics, etiologies and outcomes between anti-RNApol3 positive (RNApol3+) and negative (RNApol3-) myocarditis, we used the AMPHIBIA myocarditis cohort. The AMPHIBIA (Acute Myocarditis Registry With Prognostic, Histologic, Immunologic, Biological, Imagine and Clinical Assessment, NCT04844151) study is an ambispective cohort study including all CMR or biopsy-proven acute myocarditis cases admitted to the Cardiology Institute of La Pitié-Salpêtrière Hospital from 2008 to 2022 (retrospective group) and from 2022 to 2042 (prospective group). To constitute the control group, we selected all the patients from AMPHIBIA retrospective cohort with complete available data (2008–2019) and a negative antinuclear antibodies screening (or, if positive, with a negative RNApol3 antibodies screening).

### Statistical analyses

Continuous variables are presented as median with interquartile range (IQR) [25–75] and compared using Wilcoxon’s signed-rank tests. Categorical variables are expressed as *n* (%) and compared using chi-square tests or Fisher exact tests when appropriate. A *p*-value < 0.05 was considered statistically significant. We analyzed 6-month overall survival between RNApol3 positive and RNApol3 negative groups using Kaplan-Meier estimates. We assessed the main predictors of 6-month survival with a univariable Cox proportional hazards model, employing a variety of variables such as age, gender, body mass index (BMI), cause of myocarditis, and others. Due to the low number of deaths in both groups, multivariable Cox models were not performed. Analyses were performed using R v3.6.2 (R Core Team (2021). R: A language and environment for statistical computing. R Foundation for Statistical Computing, Vienna, Austria).

### Ethical considerations

This study was conducted in accordance with the declaration of Helsinki and utilized the database registered at the Commission Nationale de l’Informatique et des Libertés (CNIL, registration no. 1950673). In agreement with the ethical standards of our hospital’s institutional review board, the Committee for the Protection of Human Subjects, and French law, written informed consent was not needed for the analysis of demographic, physiological and hospital-outcome data, as this observational study did not modify existing diagnostic or therapeutic strategies. However, patients and/or their relatives were informed of their anonymous inclusion in the study.

## Results

### Patients’ general characteristics

**Fig. 1:**
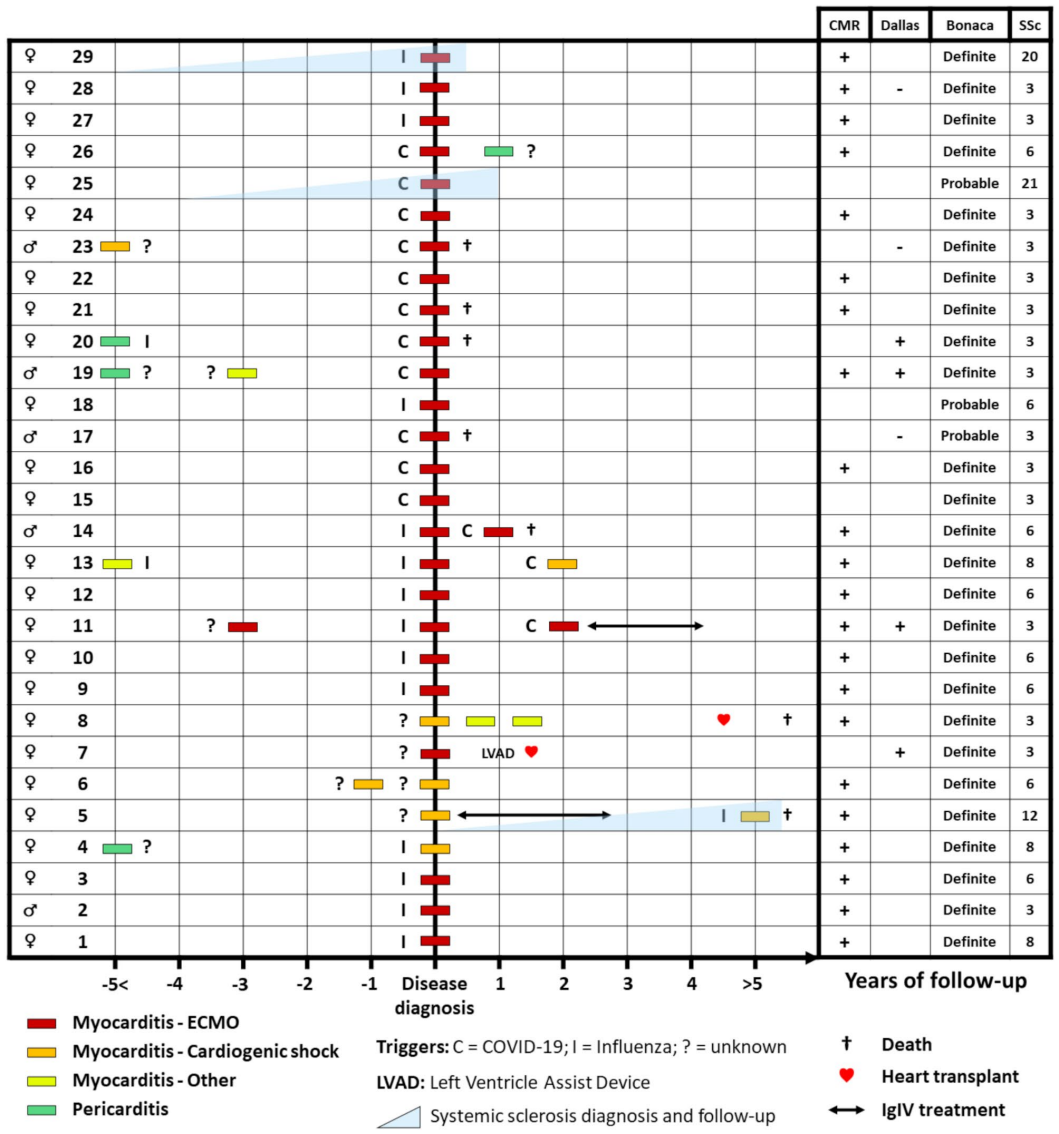
Swimmer Plot Reporting Clinical History and Viral Triggers Of The 29 Patients. Clinical history before and after diagnosis of the 29 patients with their associated viral triggers, management outcome of each episode. Abbreviations: C, COVID-19 as a trigger; CMR, cardiac magnetic resonance; ECMO, extracorporeal membrane oxygenation; I, influenza virus as a trigger; IgIV, intravenous immunoglobulins; SSc, ACR-EULAR systemic sclerosis score

From January 2013 to June 2023, 29 patients (female 83%, mean ± SD age at admission 39 ± 12 years) had 47 episodes of myocarditis and tested positive for anti-RNApol3 antibodies. Clinical and biological data were sufficient to describe only 36 episodes (of which 33 ICU episodes). Their characteristics are reported in Table 1. Every patient was admitted to the ICU at least once for a severe episode. Eleven patients (38%) experienced ≥ 1 relapse, with a median time of 3 years from the first to the second episode. The triggers in the 29 patients were: influenzae virus (55%), SARS-CoV-2 virus (48%), and flu-like syndrome of unknown cause (10%). Before the myocarditis episode, two patients had a prior diagnosis of anti-RNApol3 autoantibodies-associated systemic sclerosis. These two patients exhibited sclerodactyly, skin thickening of the arms, arthralgia, Raynaud’s phenomenon, and esophageal dysmotility. Two other patients met the criteria for mild systemic sclerosis after their myocarditis episode. The remaining patients did not meet systemic sclerosis criteria at myocarditis diagnosis or during follow-up, even though 48% had mild symptoms suggestive of the disease. Among the 20 patients who had a subsequent immunodot assay, all remained positive after a median time of 9 months. After a median follow-up of 28 months, 22 (76%) patients were alive. Two patients underwent cardiac transplantation; one was bridged to transplant after receiving a left ventricular assist device. The median left ventricle ejection fraction (LVEF) at the last follow-up was 60 [55–60] %. Over the 7 deceased patients (24%), 6 died during a fulminant myocarditis episode, and one had a hypoxic cardiac arrest 12 months after cardiac transplantation. The detailed description of each patient’s evolution and outcome is reported in Fig. 1.

**Table 1:** Characteristics of the 29 patients with RNApol3-Associated Fulminant myocarditis

Variables	*n* = 29
Total number of episodes	47
Age at disease onset, years	39 ± 12
Female	24 (83)
BMI, kg/m²	25.6 ± 5.4
Clinical findings	
At least one episode of myocarditis	29 (100)
At least one episode of isolated pericarditis	4 (14)
Patients with at least one relapse	11 (38)
Time from disease onset to first relapse, years	3 [1–6]
Total number of relapses	18
At least one intensive care unit admission	29 (100)
At least one influenza-related episode^1^	16 (55)
At least one COVID-19-related episode	14 (48)
Only flu-like syndrome-related episodes	3 (10)
Laboratory findings	
Antinuclear antibodies positivity	29 (100)
Anti-RNA polymerase III positivity	29 (100)
Time from first to last positivity^3^, month	9 [2–26]
Autoantibodies negativation^3^	0 (0)
Other autoantibodies positivity^4^	5 (17)
Systemic sclerosis findings	
Definite SSc at myocarditis onset	2 (7)
Definite SSc during follow-up	2 (7)
Raynaud’s phenomenon	14 (48)
Abnormal nailfold capillaries	4/12 (33)
Skin thickening	4 (14)
PAH and/or ILD	1 (3)
Telangiectasia	0 (0)
Fingertip lesions	0 (0)
ACR-EULAR score	3 [3–6]
Relapse-preventing specific treatment	2 (7)
Intravenous immunoglobulins	2 (7)
Outcomes	
Duration of follow up, months	28 [2–72]
Alive at last follow up^5^	22 (76)
Cardiac transplantation	2 (7)
Left ventricular assist device	1 (3)
LVEF at last follow up, %	60 [55–60]
Troponin at last follow up^6^, ng/L	9 [3–21]

Abbreviations: ACR-EULAR, American College of Rheumatology – European League Against Rheumatism; BMI, body mass index; COVID-19, coronavirus associated disease 2019; ILD, interstitial lung disease; LVEF, left ventricular assist device; PAH, pulmonary arterial hypertension; RNA, ribonucleic acid; SSc, systemic sclerosis. Continuous variables are expressed as mean ± SD or median [interquartile range 25–75]; categorical variables are expressed as No. (%)

^1^If the 36 documented episodes are considered independently, influenza related *n* = 15 (42%), SARS-CoV-2 related *n* = 14 (39%), unknown virus but flu-like symptoms *n* = 7 (19%)

^3^Data available for 21 patients

^4^Anti-histone *n* = 1, anti-centromere *n* = 2 and anti-ribonucleoprotein *n* = 1, anti-citrullinated protein antibodies = 1

^5^Six patients died of fulminant myocarditis; 1 patient died of cardiac arrest after cardiac transplantation

^6^Data available for 22 patients

### Cardiac and pericardial characteristics

**Fig. 2:**
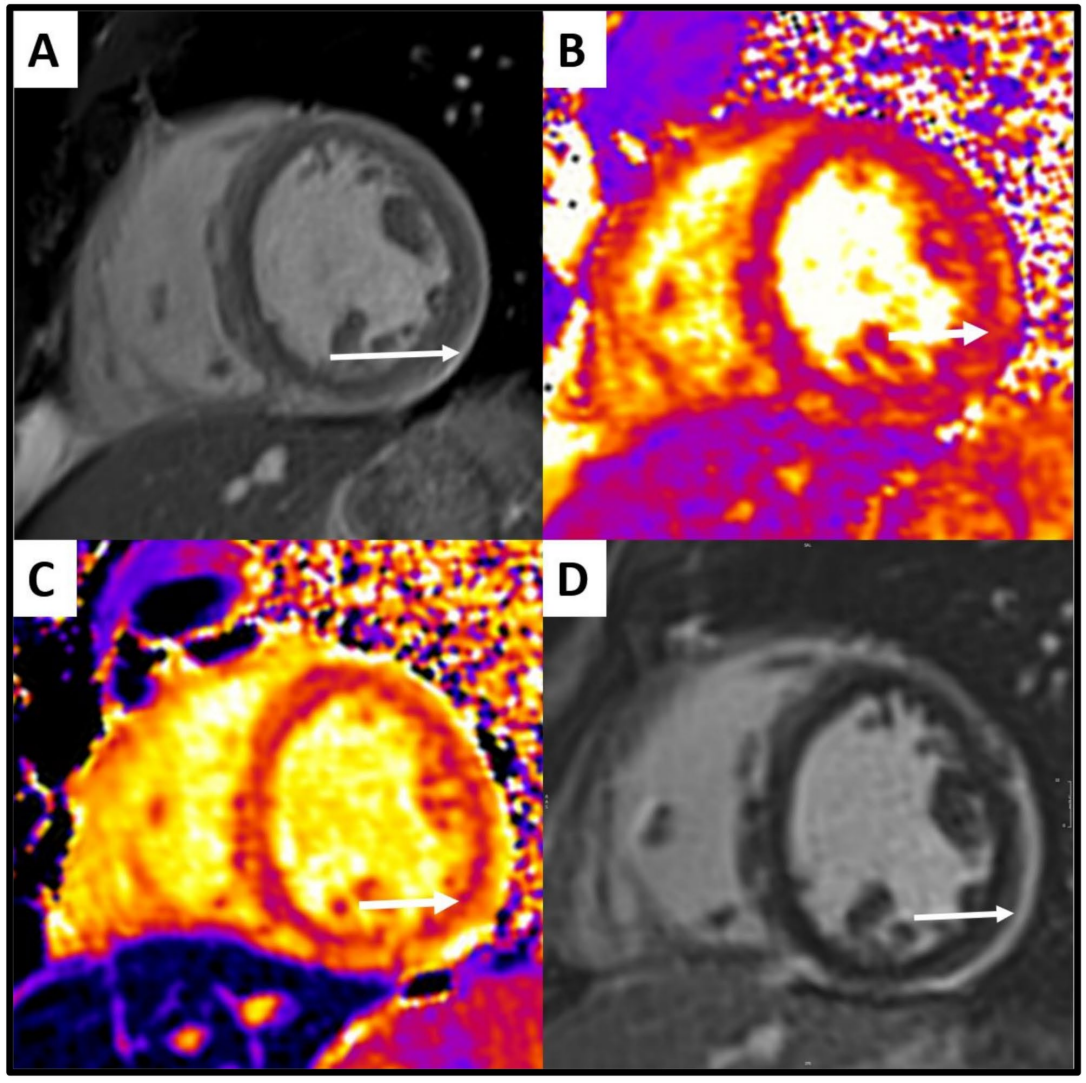
CMR Imaging Findings Of A 39-Years-Old Woman With An Acute RNA Polymerase 3 + Myocarditis. A/Short axis (SA) cine SSFP sequence showed a septal, inferior and lateral early gadolinium enhancement(arrow). B/SA native T1 mapping demonstrated an increased T1 relaxation time in the inferolateral region (1260 ms)(arrow). C/T2 mapping showed an increased T2 relaxation time (68 ms)(arrow). D/The corresponding LGE sequence found a subepicardial LGE in the lateral, inferior and inferoseptal segment(arrow)

The characteristics of the 36 documented episodes are presented in Table 2. The most frequent symptoms were cardiogenic shock (92%), chest pain (89%), and fever (75%). Electrocardiographic abnormalities were found 50% of cases, mostly repolarization changes. The highest median troponin value was 82 [19–360] times the upper limit of normal. Echocardiography at admission revealed wall motion abnormalities (92%), pericardial effusion (72%), and left ventricular hypertrophy (36%). The median lowest LVEF was 10 [5-10] %. Six patients underwent coronary angiography, all of which were normal. Twenty-two patients underwent 26 cardiac magnetic resonance (CMR) studies. CMR was performed on a median 17 [9–33] days after admission. Early gadolinium enhancement was present in 48% of cases while late gadolinium enhancement was present in 67%, mostly with subepicardial localization (Fig. 2). Oedema was present in 69% of cases and fibrosis in 63%. Overall, myocarditis according to Lake Louise criteria [12] was confirmed in 46% of the CMR (Supplemental Table 1). Endomyocardial biopsy was performed in 7 patients, confirming the diagnosis of myocarditis histologically in 5. Overall, according to the Bonaca myocarditis criteria, 89% of patients had at least one episode classified as a definite myocarditis and 10% as a probable myocarditis.

**Table 2:** Clinical and cardiac findings during the 36 Episodes1 of fulminant myocarditis

Variables	*n* = 36
Clinical findings	
Cardiogenic shock	33 (92)
Chest pain	32 (89)
Fever	27 (75)
Abdominal pain	13 (36)
Syncope	13 (36)
Respiratory failure	12 (33)
Cardiac arrest	5 (14)
Electrocardiographic changes	18 (50)
Repolarization changes	15 (42)
Rhythm disorders	2 (6)
Conduction disorders	1 (3)
Laboratory findings^2^	
Troponin highest value, fold over ULN	82 [22–360]
Creatine kinase highest value, U/L	3566 [920-14518]
Echocardiographic findings	
Wall motion abnormalities	33 (92)
Left ventricular hypertrophy	13 (36)
Lowest LVEF, %	10 [5–10]
Pericardial effusion	26 (72)
Coronary angiography	6 (17)
No significant coronary stenosis	6/6 (100)
Cardiac magnetic resonance	
Endomyocardial biopsy	7 (19)
Myocarditis on biopsy	5/7 (71)
Bonaca myocarditis^3^ criteria	
Definite	30 (83)
Probable	5 (14)
Possible	1 (3)

Abbreviations: LVEF, left ventricular ejection fraction; ULN, upper limit of normal

Continuous variables are expressed as median [interquartile range 25–75]; categorical variables are expressed as No. (%)

^1^Over the 47 patients reported episodes, only 36 with sufficient data available are herein reported

^2^Data available: troponin *n* = 34; CK *n* = 32

^3^Ref [6]

### In ICU evolution and outcome

Thirty-three (92%) episodes required ICU admission for a median duration of 10 days, with a median day-0 SOFA of 12 [8-15]  (Table 3). In-ICU organ failure treatments included dobutamine (94%), veno arterial extracorporeal membrane oxygenation (VA-ECMO, 85%), vasopressors (79%), mechanical ventilation (76%) and renal replacement therapy (30%). Pericardiocentesis was required for 38% of the episodes. Six patients died during their ICU stay due to fulminant myocarditis.

**Table 3:** Characteristics of the 33 episodes requiring Intensive Care Unit Admission

Variables	*n* = 33
Time from symptoms onset to ICU admission, days	5 [4–6]
Time in ICU, days	10 [6–16]
Day-0 SOFA score	12 [8–15]
Day-0 SAPS II score	51 [42–72]
Laboratory findings at ICU admission^1^	
White blood cells count, G/L	9.7 [7.2–15.3]
Haemoglobin level, g/dL	14 [11.3–16.1]
Proteinemia, g/L	56 [44–63]
C-reactive protein, mg/L	7 [5–12]
Procalcitonine, µg/L	0.1 [0.06–0.42]
Arterial lactate, mmol/L	6 [3–8]
In-ICU organ failure treatments	
Dobutamine	31 (94)
Dobutamine duration, days	7 [5–11]
Vasopressors	26 (79)
Intra-aortic balloon counterpulsation	18 (55)
Extracorporeal membrane oxygenation	28 (85)
ECMO duration, days	6 [5–9]
Mechanical ventilation	25 (76)
Renal replacement therapy	10 (30)
Pericardial drainage	11 (33)
Outcomes	
Left ventricular assist device	1 (3)
Cardiac transplantation	2 (6)
In-ICU mortality	6 (18)

Abbreviations: CMR, cardiac magnetic resonance; ICU, intensive care unit; SAPS II, simplified acute physiology score; SOFA, sequential organ failure assessment. Continuous variables are expressed as median [interquartile range 25–75]; categorical variables are expressed as No. (%)

^1^Data available: leukocytes *n* = 31, hemoglobin *n* = 31, proteinemia *n* = 29, C-reactive protein *n* = 21, procalcitonin *n* = 27 and arterial lactate *n* = 31

### Comparison between RNApol3 + and RNApol3-myocarditis

**Fig. 3:**
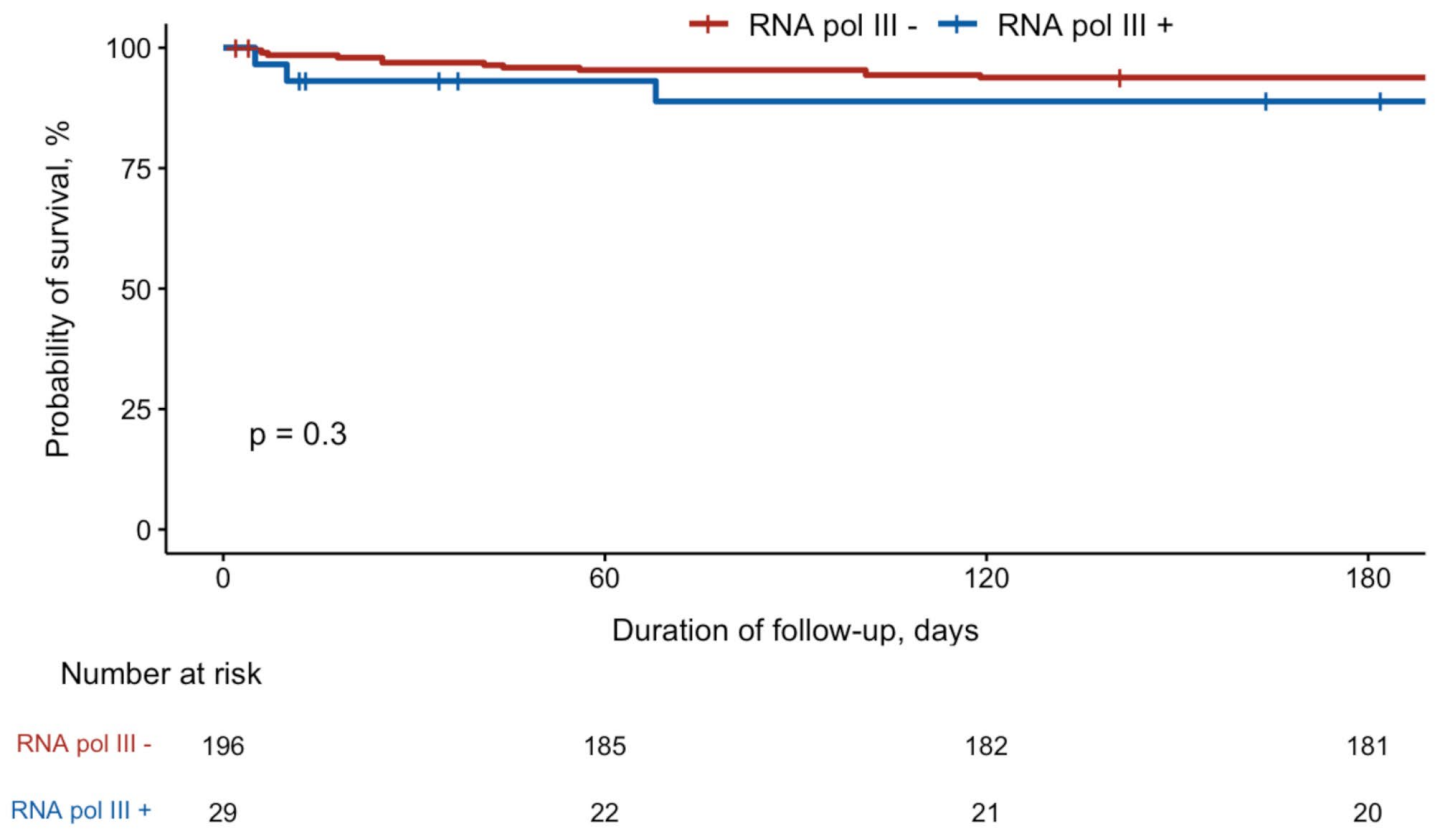
Kaplan–Meier estimated probabilities of 6-month survival for RNApol3 + vs. RNApol3-myocarditis. Survival probabilities were compared using log-rank test

We compared the characteristics, etiologies and outcomes of RNApol3 + myocarditis to a historical cohort of 196 RNApol3-myocarditis cases admitted to our institution from 2008 to 2019 (Table 4). RNApol3 + patient were more frequently women (83% vs. 28%) and more often exhibited a fulminant course, as evidenced by a lower LVEF nadir (5% vs. 52%) and more frequent use of organ failure treatments (dobutamine 90% vs. 21%, vasopressors, 79% vs. 24%, ECMO 86% vs. 15%). Pericardial effusion (81% vs. 24%) and pericardiocentesis (38% vs. 2%) were more frequent in RNApol3 + myocarditis. RNApol3 + myocarditis was more likely to be caused by a proven viral infection (90% vs. 4%, *p* < 0.001), especially influenza virus (52% vs. 1%). The frequency of myocarditis relapse was higher in RNApol3 + myocarditis (38% vs. 8%). The 6-month mortality for RNApol3 + vs. RNApol3-myocarditis was 10% vs. 5% (*p* = 0.2). The Kaplan–Meier estimated probabilities of 6-month survival, in Fig. 3 was not significatively different between RNA pol3 + and RNPApol3-patients (*p* = 0.3). The univariate Cox proportional model of factor associated with 6-month mortality is presented in Supplemental Table 2. Main factors associated with 6-month mortality included age, arrhythmia, conduction disorders, LVEF, sub-aortic VTI, troponin as well as autoimmune/inflammatory and toxic/genetic causes of myocarditis. Compared to the 6-month mortality of myocarditis of unknown cause, RNApol3 + myocarditis showed only a nonsignificant trend towards higher mortality.

**Table 4:** Comparison of characteristics, etiologies and outcomes between RNApol3 + and RNApol3-myocarditis

Variables		RNApol3 + myocarditis	RNApol3-myocarditis	*p*-value^2^
	*n*	*n* = 29^*1*^	*n* = 196^*1*^	
Age, years	225	37 [33–43]	31 [24–45]	0.1
Female	225	24 (83)	55 (28)	< 0.001
BMI, kg/m^2^	224	23.9 [22.3–28.7]	23.8 [21.4–27.0]	0.5
Clinical findings				
Fever	225	22 (76)	58 (30)	< 0.001
Chest pain	225	25 (86)	150 (77)	0.2
Pre-admission cardiac arrest	225	4 (14)	18 (9)	0.5
Arrhythmia	225	2 (7)	11 (6)	0.7
Conduction disorders	225	1 (3)	11 (6)	> 0.9
Repolarization disorder	225	13 (45)	120 (61)	0.09
LVEF lowest value, %	225	5 [5–10]	52 [25–60]	< 0.001
Sub-aortic VTI lowest value, cm/s	182	5 [4–6]	18 [14–21]	< 0.001
Pericardial effusion	222	22 (81)	47 (24)	< 0.001
Laboratory findings				
Arterial lactate, mmol/L	82	6 [3–8]	2 [1–3]	< 0.001
C-reaction protein, mg/L	193	8 [5–15]	28 [6–82]	0.02
Procalcitonine, µg/L	114	0.2 [0.1–0.5]	0.2 [0.1–0.7]	0.6
Troponin highest value, ng/L	224	1,144 [387-5,028]	665 [185-1,991]	0.06
Troponin highest value, fold over ULN	224	93 [43–368]	73 [22–171]	0.1
Antinuclear autoantibodies	225	29 (100)	63 (32)	< 0.001
RNApol3 autoantibodies	225	29 (100)	0 (0)	< 0.001
Myocarditis etiology				
Unknown	225	0 (0)	63 (32)	< 0.001
Suspected or proven infection	225	29 (100)	94 (48)	< 0.001
Flu-like syndrome	225	29 (100)	86 (44)	< 0.001
Proven viral infection	225	26 (90)	8 (4)	< 0.001
Flu or COVID-19	225	26 (90)	3 (1)	< 0.001
Flu	224	15 (52)	3 (1)	< 0.001
Epstein-Barr virus	224	0 (0)	3 (1)	> 0.9
Parvovirus B19	224	0 (0)	1 (1)	> 0.9
Bacteria	225	0 (0)	4 (2)	> 0.9
Immunological myocarditis				
Autoimmune/inflammatory diseases	225	0 (0)	24 (12)	0.05
Eosinophilic myocarditis	225	0 (0)	4 (2)	> 0.9
Giant-cell myocarditis	225	0 (0)	3 (1)	> 0.9
Toxic myocarditis	225	0 (0)	6 (3)	> 0.9
Genetic cardiomyopathy	225	0 (0)	2 (1)	> 0.9
Organ failure treatments				
Dobutamine	225	26 (90)	42 (21)	< 0.001
Norepinephrine	225	23 (79)	47 (24)	< 0.001
Mechanical ventilation	225	23 (79)	32 (16)	< 0.001
Renal replacement therapy	225	9 (31)	14 (7)	< 0.001
Extracorporeal membrane oxygenation	225	25 (86)	30 (15)	< 0.001
Intra-aortic conterpulsation	225	16 (55)	15 (8)	< 0.001
Myocarditis treatments				
Pericardiocentesis	225	11 (38)	4 (2)	< 0.001
Corticosteroids	225	5 (17)	32 (16)	> 0.9
Intravenous immunoglobulins	225	5 (17)	12 (6)	0.05
Any immunosuppressant	225	0 (0)	22 (11)	0.09
Outcomes				
Relapse	223	11 (38)	15 (8)	< 0.001
Heart transplant	225	2 (7)	2 (1)	0.08
Day-90 mortality	225	3 (10)	9 (5)	0.2
6-month mortality	225	3 (10)	12 (6)	0.4

Abbreviations: BMI, body-mass index; LVEF, left ventricular ejection fraction; ULN, upper limit of the normal; RNApol3, RNA polymerase III autoantibodies; VTI, velocity time integral. Continuous variables are expressed as median interquartile range (IQR) [25–75] and compared with Wilcoxon’s signed-rank tests. Categorical variables are expressed as *n* (%) and compared with chi-square tests or Fisher exact tests when appropriate

## Discussion

Anti-RNApol3 autoantibodies-associated fulminant myocarditis is a newly described disease. Herein, we further expand the knowledge on its clinical spectrum and outcomes. Our work highlights several striking findings: a marked female predominance (opposite to the usual myocarditis gender ratio [14]); a very frequent fulminant presentation of myocarditis; the prevalence of RNA viruses as triggers; the high frequency of pericardial effusion requiring drainage; the high risk of relapse facing another RNA virus; and the few patients with definite systemic sclerosis despite many exhibiting systemic sclerosis stigmata. This entity establishes a new link between infection and autoimmunity. Its pathophysiology remains unknown and warrants further investigation.

Anti-RNApol3 autoantibodies are known to be associated with severe systemic sclerosis with diffuse cutaneous involvement, scleroderma renal crisis and cancer [15–17]. Their presence is a strong predictive factor of progression from Raynaud’s phenomenon to definite systemic sclerosis [18]. While most patients in our series did not meet the criteria for systemic sclerosis, Raynaud’s phenomenon, mild sclerodactyly and gastrointestinal reflux were frequent. Initially, we thought RNApol3 autoantibodies-associated myocarditis and systemic sclerosis did not overlap. However, recently, two patients were admitted to our department with RNA virus-related fulminant myocarditis and had a long-standing diagnosis of anti-RNApol3 autoantibodies positive systemic sclerosis. Therefore, the presence of these rare autoantibodies seems to be associated with both diseases. Conversely, this also implies that anti-RNApol3-positive systemic sclerosis might be at higher risk of RNA virus-associated fulminant myocarditis. This association has never been previously described, likely due to the rarity of both diseases. Noteworthy, no patient fulfilled the criteria for scleroderma renal crisis. Anti-RNA polymerase III antibodies were confirmed during each episode and periodically between episodes in all patients following their initial identification. Further investigations on the frequency of viral myocarditis in systemic sclerosis patients are therefore needed.

We are considering the appropriate approach for systemic sclerosis patients positive for RNApol3 autoantibodies. The risk of an initial episode of fulminant myocarditis in this context is unknown. While the literature suggests a very low risk of fulminant myocarditis recurrence [19], we have observed cases of recurrent fulminant myocarditis triggered by both influenza and SARS-CoV-2 viruses in patients with RNApol3 autoantibodies. One patient even experienced three episodes of VA-ECMO-assisted-fulminant myocarditis within 5 years, all triggered by RNA viruses. Recent myocarditis theoretically contraindicates SARS-CoV-2 vaccination [20]. However, among the 23 patients alive at the end of follow-up in our series, 8 were vaccinated against SARS-CoV-2 without experiencing any adverse effects or relapses. One patient developed her first episode of SARS-CoV-2-related myocarditis 4 days after COVID-19 vaccination, but she also tested positive for SARS-CoV-2 by nasopharyngeal PCR upon ICU admission. The benefit-risk ratio of vaccination among these patients should be carefully assessed, but it appears to favor vaccination. One patient received monthly intravenous immunoglobulins (following the third episode of fulminant myocarditis requiring VA-ECMO) and there have been no relapses observed after three years. However, longer follow-up is necessary.

Systemic sclerosis is the autoimmune condition with the highest mortality among all rheumatic diseases. Its therapeutic management includes low-dose corticosteroids (as high doses can trigger scleroderma renal crisis), immunosuppressants for diffuse cutaneous and internal organ involvement (e.g., mycophenolate mofetil, cyclophosphamide, tocilizumab), vasodilators for pulmonary arterial hypertension, and calcium channel blockers for Raynaud’s phenomenon and digital ulcers. There are currently no clear guidelines for managing chronic cardiac involvement in systemic sclerosis. Whether any of these treatments could be effective in the management or prevention of RNApol3-associated myocarditis remains unknown and warrants further investigation. The pathophysiology of this disease remains elusive. RNApol3 autoantibodies could potentially be pathogenic or may reflect an autoimmune process with an unknown mechanism. While several potential cardiac autoantigens have been described in fulminant myocarditis, no clear association has been established between RNApol3 and cardiomyocytes [2]. The presence of autoantibodies targeting cytokines has been noted in various infectious diseases: anti-interferon-α antibodies in severe SARS-CoV-2 infection [21]; anti-interferon-γ antibodies in tuberculosis [22] and anti-IL17A/IL-17 F/IL-22 in chronic mucocutaneous candidiasis [23]. RNApol3 however is not a cytokine and is located in nucleus of the eukaryote cells, a site traditionally considered inaccessible to autoantibodies [24]. Nevertheless, recent data suggest that certain autoantibodies may penetrate cells and reach their target [25]. Mutations affecting RNApol3 subunits can results in immunodeficiency and increased susceptibility to viral infections [10]. Notably, RNApol3 is recognized as a viral DNA sensor, transcribing DNA viral genomes into RNA, which in turn induces of type-I IFN production. Recent findings also indicate that RNApol3 may act as a sensor for RNA viruses [26]. Further translational investigations are now imperative to unravel the pathophysiology of this emerging disease.

This study has some limitations that deserve mentioning. First, the external validity is limited by its monocentric and retrospective nature. Notably, as a high-volume ECMO center, there might be a selection bias towards including the most severe patients. Second, while being the largest series of anti-RNApol3 autoantibodies-related fulminant myocarditis cases, the sample size remains small, limiting the study’s power. Third, due to the data availability, the myocarditis control cohort did not include the years 2020–2022, precluding the comparison of COVID-19-related myocarditis between the two groups. The differing timeframes between the two groups may have introduced a selection bias. However, the control cohort was recruited over a lengthy period of 10 years, with a 7-year overlap with the RNApol3 + cohort. Lastly, we chose to compare anti-RNA polymerase III myocarditis to the entire AMPHIBIA cohort, rather than restricting the comparison to myocarditis cases admitted to the ICU. Anti-RNA polymerase III myocarditis cases could be included even if they were not admitted to the ICU. Notably, all patients in this group experienced at least one ICU admission during their disease course, although not all episodes required intensive care. We demonstrate that myocarditis associated with anti-RNA polymerase III antibodies represents a subgroup of myocarditis frequently fulminant.

## Conclusion

Anti-RNApol3 autoantibodies-associated fulminant myocarditis is an emerging disease linking autoimmunity and infection. While its pathophysiology remains elusive, it should be recognized as a unique cause of acquired, pathogen-specific, organ-specific immunodeficiency. Anti-RNApol3 autoantibodies should be screened in all cases of fulminant myocarditis, especially in young women infected by RNA viruses. The risk of fulminant myocarditis in RNApol3 autoantibodies-positive systemic sclerosis needs further investigation.

## Electronic supplementary material

Below is the link to the electronic supplementary material.

Supplementary Material 1

## Data Availability

None.

## Abbreviations

ACR-EULAR: American College of Rheumatology - European League Against Rheumatism
CMR: Cardiac magnetic resonance
DNA: Deoxyribonucleic Acid
ECMO: Extracorporeal Membrane Oxygenation
ELISA: Enzyme-linked Immunosorbent Assay
ICU: Intensive Care Unit
IFN: Interferon
IL: Interleukin
LVEF: Left Ventricular Ejection Fraction
MRI: Magnetic Resonance Imaging
RIG-I: Retinoic acid-inducible gene-I
RNA: Ribonucleic Acid
RNApol3: RNA-polymerase III
SAPS II: Simplified Acute Physiology Score II
SARS-CoV-2: Severe Acute Respiratory Syndrome Coronavirus 2
SSc: Systemic Sclerosis
SOFA: Sequential Organ Failure Assessment score
TNF: Tumor Necrosis Factor
