# Supplementary material for: Anti-RNApol3-Associated myocarditis: an emerging disease linking autoimmunity and infection
Source: Ann Intensive Care. 2025 Mar 24;15:38. doi: 10.1186/s13613-025-01443-1 (PMC11930900; doi:10.1186/s13613-025-01443-1)
Supplement: Supplementary file 1 — Supplementary Material 1 [file 13613_2025_1443_MOESM1_ESM.docx]

| **Supplemental Table 1. Detailed Findings Of The 26 Cardiac Magnetic Resonance Imaging** | |
| --- | --- |
| **Variables** | **n=26** |
| Time from admission to CMR, days | 17 [9-33] |
| Early gadolinium enhancement | 11/23 (48) |
| Late gadolinium enhancement | 16/24 (67) |
| Type of LGE |  |
| Transmural | 1/23 (4) |
| Subepicardial | 14/23 (61) |
| Absent | 7/23 (30) |
| Number of LGE | 2 [0-3.5] |
| LVEF, % | 54 [50-58] |
| RVEF, % | 54 [51-59] |
| Fibrosis | 15/24 (63) |
| Oedema | 18 (69) |
| Pericardial effusion | 7 (27) |
| Pericarditis | 4 (15) |
| Myocarditis according to Lake Louise criteria^1^ | 12 (46) |
| Abbreviations: CMR, cardiac magnetic resonance; LVEF, left ventricular ejection fraction; RVEF, right ventricular ejection fraction; LGE, late gadolinium enhancement. Continuous variables are expressed as median [interquartile range 25-75]; categorical variables are expressed as No. (%).  ^1^Ref [7] | |

| **Supplemental Table 2. Univariate Cox Proportional Analysis of 6-month Mortality-Associated Factor** | | | |
| --- | --- | --- | --- |
| **Variables** | **n** | **HR** **(95% CI)***^1^* | **p-value** |
| Female | 225 | 1.72 [0.64-4.78] | 0.3 |
| Age, years | 225 | 1.05 [1.02-1.08] | 0.001 |
| BMI, kg/m^2^ | 224 | 1.02 [0.94-1.12] | 0.6 |
| Clinical findings |  |  |  |
| Fever | 225 | 0.94 [0.23-2.75] | 0.9 |
| Chest pain | 225 | 0.39 [0.14-1.09] | 0.07 |
| Arrhythmia | 225 | 21.0 [7.55-58.5] | <0.001 |
| Conduction disorders | 225 | 4.99 [1.41-17.7] | 0.01 |
| Repolarization disorder | 225 | 1.89 [0.60-5.93] | 0.3 |
| LVEF lowest value, % | 225 | 0.94 [0.90-0.97] | <0.001 |
| Sub-aortic VTI lowest value, cm/s | 182 | 0.90 [0.83-0.98] | 0.01 |
| Laboratory findings |  |  |  |
| Arterial lactate, mmol/L | 82 | 1.05 [0.87-1.26] | 0.6 |
| C-reaction protein, mg/L | 193 | 1.00 [0.99-1.01] | 0.8 |
| Procalcitonine, µg/L | 114 | 1.00 [0.96-1.04] | 0.9 |
| Troponin highest value, fold over ULN | 224 | 1.00 [1.00-1.00] | <0.001 |
| Myocarditis etiology | 225 |  |  |
| Unknown |  | — |  |
| RNApol3-associated myocarditis |  | 3.7 [0.62-22.3] | 0.09 |
| Autoimmune/inflammatory diseases |  | 5.3 [1.03-27.4] | 0.03 |
| Suspected or proven infection |  | 0.32 [0.03-3.5] | 0.7 |
| Toxic or genetic |  | 18.8 [3.43-103] | 0.02 |
| Abbreviations: BMI, body-mass index; HR, hazard ration; LVEF, left ventricular ejection fraction; ULN, upper limit of the normal; RNApol3, RNA polymerase III autoantibodies; VTI, velocity time integral. | | | |
